# Spondyloarthritis mass cytometry immuno-monitoring: a proof of concept study in the tight-control and treat-to target TiCoSpA trial

**DOI:** 10.1007/s10067-023-06637-1

**Published:** 2023-06-12

**Authors:** Hester Koppejan, Guillaume Beyrend, Marjolijn Hameetman, Tamim Abdelaal, René E.M. Toes, Floris A. van Gaalen

**Affiliations:** 1 Department of Rheumatology, Leiden University Medical Center, PO box 9600 (Zone C1-R), Albinusdreef 2, 2233 ZA Leiden, The Netherlands; 2 Department of Immunology, Leiden University Medical Center, Leiden, The Netherlands; 3 Flow Core Facility, Leiden University Medical Center, Leiden, The Netherlands; 4 Department of Radiology, Leiden University Medical Center, Leiden, Leiden, The Netherlands; 5 Faculty of Engineering, Systems and Biomedical Engineering Department, Cairo University, Cairo, Egypt; 6 Delft Bioinformatics Lab, Delft University of Technology, Delft, The Netherlands

**Keywords:** Axial spondyloarthritis, CyTOF, Cytometry by time of flight, Immuno-monitoring, Mass cytometry, NCT03043846, Spondyloarthritis, Tight-control, Treat-to-target

## Abstract

**Objective:**

Mass cytometry (MC) immunoprofiling allows high-parameter phenotyping of immune cells. We set to investigate the potential of MC immuno-monitoring of axial spondyloarthritis (axSpA) patients enrolled in the Tight Control SpondyloArthritis (TiCoSpA) trial.

**Methods:**

Fresh, longitudinal PBMCs samples (baseline, 24, and 48 weeks) from 9 early, untreated axSpA patients and 7 HLA-B27^+^ controls were analyzed using a 35-marker panel. Data were subjected to HSNE dimension reduction and Gaussian mean shift clustering (Cytosplore), followed by Cytofast analysis. Linear discriminant analyzer (LDA), based on initial HSNE clustering, was applied onto week 24 and 48 samples.

**Results:**

Unsupervised analysis yielded a clear separation of baseline patients and controls including a significant difference in 9 T cell, B cell, and monocyte clusters (cl), indicating disrupted immune homeostasis. Decrease in disease activity (ASDAS score; median 1.7, range 0.6–3.2) from baseline to week 48 matched significant changes over time in five clusters: cl10 CD4 T_nai_ cells median 4.7 to 0.02%, cl37 CD4 T_em_ cells median 0.13 to 8.28%, cl8 CD4 T_cm_ cells median 3.2 to 0.02%, cl39 B cells median 0.12 to 2.56%, and cl5 CD38^+^ B cells median 2.52 to 0.64% (all *p*<0.05).

**Conclusions:**

Our results showed that a decrease in disease activity in axSpA coincided with normalization of peripheral T- and B-cell frequency abnormalities. This proof of concept study shows the value of MC immuno-monitoring in clinical trials and longitudinal studies in axSpA. MC immunophenotyping on a larger, multi-center scale is likely to provide crucial new insights in the effect of anti-inflammatory treatment and thereby the pathogenesis of inflammatory rheumatic diseases.

**Table:** 

**Key Points** • *Longitudinal immuno-monitoring of axSpA patients through mass cytometry indicates that normalization of immune cell compartments coincides with decrease in disease activity.* • *Our proof of concept study confirms the value of immune-monitoring utilizing mass cytometry.*

**Supplementary Information:**

The online version contains supplementary material available at 10.1007/s10067-023-06637-1.

## Introduction

Axial spondyloarthritis (axSpA) is a chronic, HLA-B27-associated inflammatory disease [1]. It manifests mainly in the axial joints, though patients often also suffer from enthesitis, dactylitis, psoriasis, anterior uveitis, arthritis of peripheral joints, and inflammatory bowel disease [2]. Early treatment initiation is crucial to reduce the burden of disease as, e.g., anti-inflammatory treatment using TNF inhibitors is most effective when initiated in early disease [3] also emphasizing that early diagnosis is important as sustained disease activity results in irreversible structural damage and poor functioning [4].

The pathogenesis of axSpA is incompletely understood but is thought to be multifactorial [5]. Although some key factors, including HLA-B27, have been identified, the underlying pathophysiological mechanism yet remains to be clarified. Additionally, it is difficult to obtain in situ material of patients, as the inflamed axial sites are difficult to access. Clinical studies therefore commonly use peripheral samples, including serum, peripheral blood mononuclear cells (PBMCs), or mononuclear cells isolated from synovial fluid obtained from inflamed peripheral joints. As a consequence of limited knowledge on the pathogenesis of axSpA, current treatments focus on dampening the inflammatory response to avoid further damage, and as of today, no curative treatment is available.

Mass cytometry (MC) is an antibody-based high-parameter technology which combines cytometry with inductively coupled plasma time of flight (ICP-TOF) mass spectrometry to allow investigation of >40 markers with a single-cell resolution [6]. To this end, antibodies tagged with stable heavy metal isotopes are utilized to label markers of interest, similar to flow cytometry. Cells are acquired, and the heavy metal tags are quantified on a single-cell level. This technology is suited for performing explorative immunophenotyping studies and has been used in the context of rheumatic diseases [7–14].

Longitudinal immuno-monitoring could be very valuable in context of response-to-treatment studies as it allows broad screening of the cellular immune system to track which changes can be observed following treatment initiation. Additionally, learning more about compartments (positively) affected by treatment could also enhance our knowledge of disease onset or biomarker discovery. The goal of this study was to evaluate the patient’s cellular immunophenotype changes over time after treatment initiation and to assess if MC could be valuable to extend our knowledge of axSpA pathophysiology and/or provide biomarkers to predict response to therapy. To this end, we utilized material obtained from patients participating in the “TICOSPA” trial (NCT03043846), a tight control treat-to-target clinical trial [15]. This trial focused on lowering disease activity (ASDAS score) by following a pre-specified treatment strategy based on the current international scientific recommendations for axSpA management. We set out to study freshly isolated PBMCs of patients at baseline, week 24, and week 48 to assess differences compared to healthy controls and to follow the immune compartments over time during treatment to evaluate the value of MC when investigating axSpA disease pathogenesis.

## Materials and methods

### Patient material

Heparin blood was collected from TiCoSpA study participants [15] (*n*=9) during three visits (baseline, week 24, and week 48) from the Leiden University Medical Center (LUMC), The Netherlands. All LUMC TiCoSpA participants were assigned to the “TC/T2T algorithm” and strictly followed pre-specified strategy based on the current international scientific recommendations for axSpA management. Inclusion criteria were described previously [15]. Briefly, patients had to be 18–65 years old, HLA-B27 positive, and diagnosed with axSpA by rheumatologist following the ASAS criteria for axSpA and at inclusion, and patients should have active disease defined by ASDAS≥ 2.1. In addition, patients could not have been optimally treated with NSAIDs (i.e., they could not have received two full courses of NSAIDs at a daily full dose for at least 2 weeks each), should have no contraindications to NSAIDs, should be biologic naive, and should not have received apremilast in the past 3 months. HLA-B27^+^, age-matched control subjects without axial spondyloarthritis (*n*=7), from SPACE (SPondyloArthritis Caught Early) cohort study [16] came in for a single heparin blood collection. Patient and control samples were processed according to the same protocol (described below). Studies were approved by the LUMC ethical committee, and all patients provided written informed consent. Patient characteristics are summarized in Table 1. One out of 27 patient samples could not be acquired, resulting in only 8 patient samples for week 24.

**Table 1:** Baseline patient characteristics

	TiCoSpA	Controls
	*N*=9	*N*=7
Age median (range), years	32 (19–47)	37 (22–49)
Females, *n* (%)	4 (44%)	5 (71%)
Median duration back pain (range), months	48 (3–276)	
Inflammatory back pain, *n* (%)	9 (100%)	
Good response of back pain to NSAIDs	8 (89%)	
Family history of SpA, *n* (%)	2 (22%)	
Anterior uveitis, *n* (%)	0 (0%)	
Peripheral arthritis, *n* (%)	2 (22%)	
Dactylitis, *n* (%)	0 (0%)	
Enthesitis, *n* (%)	1 (11%)	
Psoriasis, *n* (%)	0 (0%)	
IBD, *n* (%)	0 (0%)	
CRP (mg/L) median (range)	6.9 (2–29)	
Elevated CRP *n* (%)	6 (67%)	
HLA-B27 positive, *n* (%)	8 (89%)	7 (100%)
Sacroiliitis on MRI, n%	7/7 (100%)*	
Sacroiliitis on radiography, *n* (%)	3 (33%)	
ASDAS median (range)	3.3 (2.1–4.9)	
Current DMARD use, *n* (%)	0 (0%)	

*TiCoSpA* tight control in spondyloarthritis, *NSAID* nonsteroidal anti-inflammatory drug, *DMARD* disease-modifying antirheumatic drugs, *ASDAS* Ankylosing Spondylitis Disease Activity Score, *AxSpA* axial spondyloarthritis, *SpA* spondyloarthritis. MRI results missing in two patients

### Sample processing

Freshly obtained heparin blood was processed within a few hours upon collection: PBMCs were isolated through Ficoll-Paque gradient centrifugation followed by a viability stain using Cell-ID intercalator-103^Rh^ (Fluidigm, South San Francisco, CA, USA). Next, cells were fixed (1.85% Formaldehyde solution; Sigma-Aldrich, Darmstadt, Germany; diluted in Maxpar PBS, Fluidigm) and stored in Maxpar Cell Staining Buffer (CSB, Fluidigm). Processed samples were stored for a maximum of 3 days in suspension.

### Mass cytometry staining and acquisition

Thirty-five pre-conjugated or self-conjugated antibodies (Maxpar X8 labeling kit at 100ug scale, Fluidigm) were used for mass cytometry staining and are listed in Supplementary Table 1. Sample staining was performed according to manufacturer’s protocol (Fluidigm). Briefly, pre-processed samples were treated with Fc-block (Human TruStain FcX, Biolegend, San Diego, CA, USA), stained for 33 surface markers, and permeabilized (manufacture’s protocol; eBioscience Foxp3/Transcription Factor Staining Buffer Set, eBioscience, San Diego, CA, USA) followed by IL-17A and Ki-67 staining. All cells were stained with Cell-ID intercalator-Iridium (191^Ir^/193^Ir^, Fluidigm) diluted in Maxpar Fix and Perm Buffer (Fluidigm) at a final concentration of 125nM and kept at 4 degrees Celsius for 48hrs.

On the day of acquisition, samples were washed and diluted into Maxpar water (Fluidigm) to obtain a final concentration of 0.75×10^6^ cells/ml and 10% v/v EQ beads (EQ Four Element Calibration Beads, Fluidigm). All samples were acquired on a Fluidigm Helios CyTOF system using the HT injector. Following acquisition, FCS were normalized within the Fluidigm acquisition software (reference EQ passport P13H2302) and concatenated if required. Technical variation was monitored through the use of an internal reference control that was included into each staining/acquisition batch (buffy coat obtained from Sanquin, The Netherlands).

### Mass cytometry data analysis

Normalized FCS files were loaded into FlowJo v10 (TreeStar, Ashland, OR, USA) for data clean up and exported into new FCS files. Newly obtained FCS files consisted solely of pre-gated single, live, CD45^+^ cells (Supplementary Figure 1). The first analysis aimed to obtain an immune profile of baseline and control samples. To this end, 11.5*10^6^ CD45^+^ cells originating from 16 samples (9 baseline and 7 controls) were sample-tagged, hyperbolic ArcSinh-5 transformed, and simultaneously subjected to dimension reduction in Cytosplore v2.2 [17]. A broad immunophenotype was obtained using a 6-level HSNE combined with Gaussian mean shift clustering (default settings). Clusters were exported as FCS files for downstream analysis using the Cytofast analysis pipeline [18], comparing baseline patients to control samples.

The clusters obtained from baseline and control samples were used to train a classifier (linear discriminant analysis, LDA) [19]. This classifier was applied to annotate the newly acquired cells (approx. 10*10^6^ in total) originating from the patients’ samples obtained at week 24 (*n*=8) and week 48 (*n*=9). Based on the Cytofast results comparing baseline and control, significantly different clusters were selected for further analysis. The frequency of these clusters in week 48 samples was compared to baseline to monitor the effect of treatment in these clusters specifically.

Statistical analysis was performed in R using Cytofast when analyzing all baseline/control clusters simultaneously. The second analysis, comparing cluster frequency of week 48 patient samples versus controls, was performed using the non-parametric Wilcoxon Rank Test in GraphPad Prism v8 (GraphPad Software, La Jolla, CA, USA). Statistical significance was considered when *p* < 0.05.

## Results

### Immunophenotyping baseline and controls results in 9 significantly different clusters

**Fig. 1:**
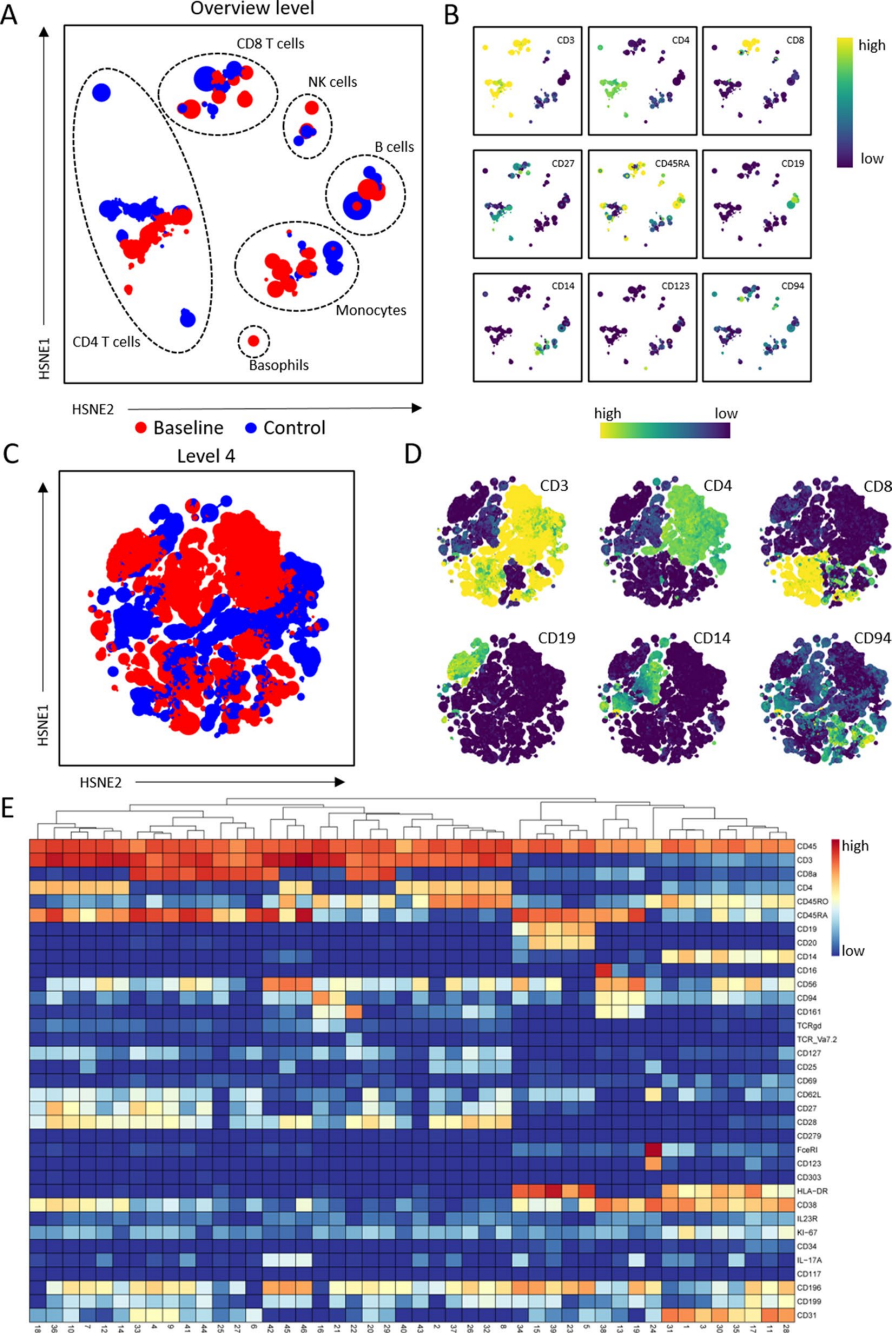
Unsupervised dimension reduction and clustering reveal the major immune cell populations in pooled samples of untreated axSpA patients and controls. **A** Overview level HSNE separated all major lineages as indicated by dashed circles (RED patients *n*=9; BLUE controls *n*=7). Solid circle shows example of differences in CD4 T cells. **B** Representative examples of marker expression as shown in **A**. **C** Zoom of total events to HSNE level showing 274*10^5^ identified landmarks (colors as in A). **D** Representative examples of marker expression as shown in **C**. **E** Heatmap of 46 clusters identified through unsupervised clustering in Cytosplore (Gaussian mean shift). Columns represent clusters, and rows represent each marker included in the analysis. Left to right: 29 T cell clusters (CD3^+^), 5 B cell clusters (CD19^+^), 3 NK cell clusters (CD56^+^CD94^+^), single pDC/basophil cluster (CD123^+^ and FcεRI^+^), and 8 monocyte clusters (CD14^+^)

**Fig. 2:**
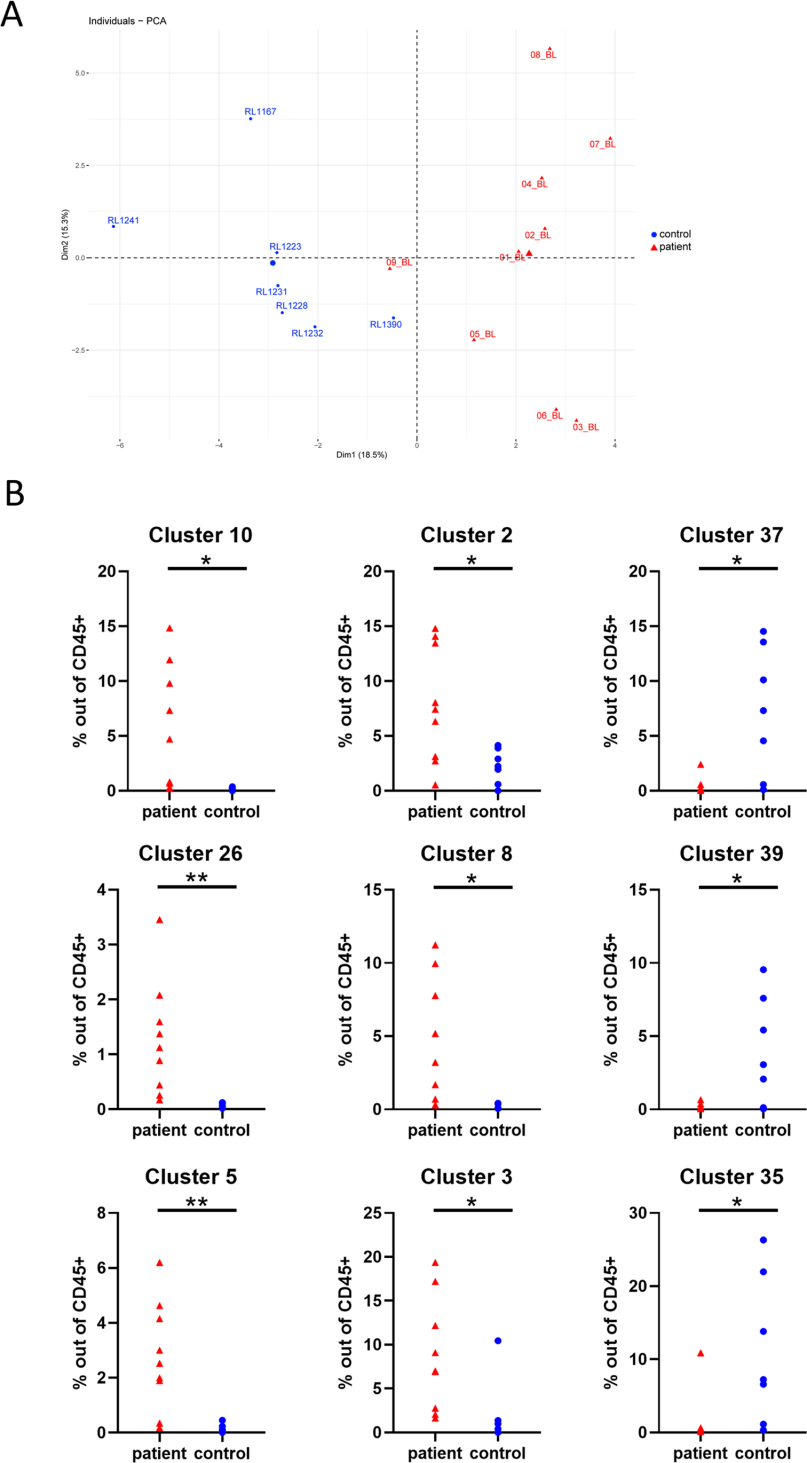
AxSpA patients and controls separate based on their mass cytometry immune profile. **A** Baseline patient and control samples separate in PCA based on 35-marker immune profile (defined by 46 Cytosplore clusters as input). **B** Frequencies of nine clusters are significantly different at baseline (red) compared to controls (blue): CD4 T_nai_ cells (cl10 *p*=0.02), CD4 T_em_ cells (cl2 *p*=0.01; cl37 *p*=0.02; cl26 *p*=0.01), CD4 T_cm_ cells (cl8 *p*=0.02), B cells (cl39 *p*=0.03; cl5 *p*=0.004), and monocytes (cl3 *p*=0.02; cl35 *p*=0.05). Cluster 10, cluster 2, cluster 26, cluster 8, cluster 5, and cluster 3 have a higher frequency in patients, whereas cluster 37, cluster 39, and cluster 35 have a higher frequency in controls. *p≤0.05; **p≤0.01

Mass cytometry (MC) was used to quantify a 35-marker panel in 16 samples (9 baseline; 7 controls). The HSNE clustering could identify major lineages, which were clearly separated, including CD4^+^/CD8^+^ T cells, NK-cells, B-cells, monocytes, and basophils (Fig. 1A, B). Discrimination between patient and controls became readily apparent as, e.g., one of the CD4 islands separated between both groups. These observations were mainly driven by CD27 expression, which was present in patients but absent in controls (Fig. 1B). Consequently, we focused on the differences at a deeper level from all the populations identified earlier and “zoomed in” within the Cytosplore software (Fig. 1C, D). This was followed by default Gaussian mean shift clustering, which resulted in 46 clusters (Fig. 1E). The majority of clusters represented T cells (29 out of 46), the remainder included 5 B cell subsets (CD19^+^), 3 NK cell subsets (CD56^+^CD94^+^), a single pDC/basophil subset (CD123^+^ and FcεRI^+^), and 8 monocyte subsets (CD14^+^). Utilizing the Cytofast analysis pipeline, we analyzed the frequency of all 46 clusters comparing baseline to control (Supplementary Figure 2A/B). Based on this input, a PCA analysis showed clear separation of patients and controls, indicating that each group has a distinct immune signature (Fig. 2A). Out of the 46 identified clusters, the frequency of 9 clusters was significantly different between baseline and controls: CD4 T_nai_ cells (cl10 *p*=0.02), CD4 T_em_ cells (cl2 *p*=0.01; cl37 *p*=0.02; cl26 *p*=0.01), CD4 T_cm_ cells (cl8 *p*=0.02), B cells (cl39 *p*=0.03; cl5 *p*=0.004), and monocytes (cl3 *p*=0.02; cl35 *p*=0.05) (Fig. 2B).

### Decrease in ASDAS coincides with normalization of the cellular immunophenotype

**Fig. 3:**
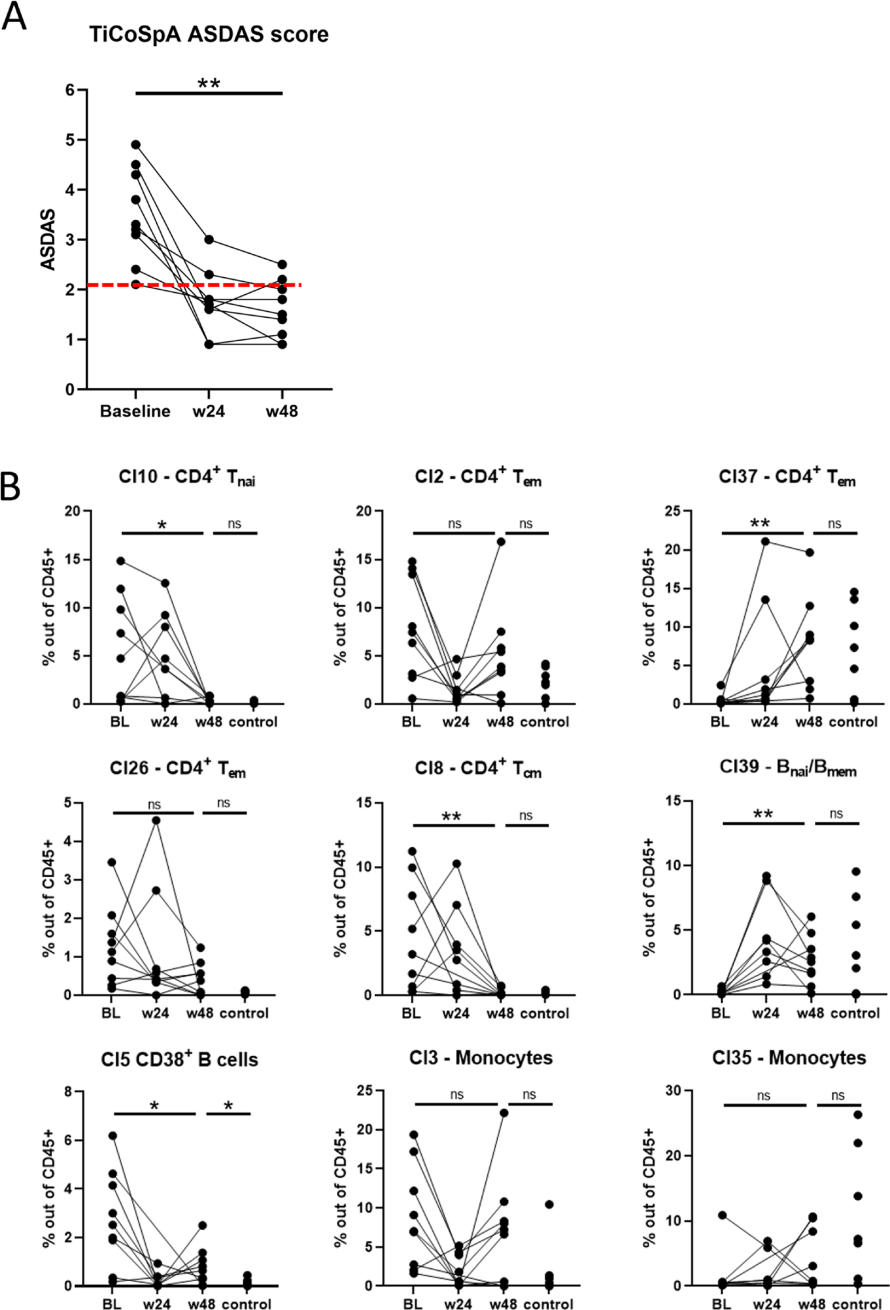
Immuno-monitoring indicated treat-to-target decreased ASDAS coincided with largely normalization of axSpA immunophenotype. **A** ASDAS was reduced following treat-to-target in all 9 patients: median decrease of 1.7 of baseline vs week 48; range 0.6–3.2; *p*=0.004. 7/9 patients achieved target ASDAS (<2.1; range 0.9–2); ASDAS of remaining two patients at week 48 was 2.5 (baseline ASDAS 4.9) and 2.2 (baseline ASDAS 4.5). Red dashed line indicates ASDAS 2.1 cut-off. **B** Frequencies of clusters of interest identified in Fig. 2B for all patient time-points and controls. 5/9 changed significantly during treatment: cl10 CD4 T_nai_ cells median 4.7% to 0.02% *p*=0.04, cl37 CD4 T_em_ cells median 0.13% to 8.28% *p*=0.008, cl8 CD4 T_cm_ cells median 3.2% to 0.02% *p*=0.008, cl39 B cells median 0.12% to 2.56% *p*=0.008, cl5 CD38^+^ B cells median 2.52% to 0.64% *p*=0.02. In 4 of these clusters (cl10, cl37, cl8, cl39), week 48 frequencies were comparable to controls. *p≤0.05; **p≤0.01

All LUMC patients enrolled in the TiCoSpA study were treated according a treat-to-target strategy following current scientific recommendations for axSpA management. At week 24, all patients received NSAID (nonsteroidal anti-inflammatory drug) treatment, and by week 48, all patients had progressed to DMARD (disease-modifying antirheumatic drug) treatment or a combination of NSAIDs and DMARDs (*n*=3). The treat-to-target strategy did manage to decrease ASDAS substantially (median decrease of 1.7; range 0.6–3.2) and significantly (*p*=0.004) in all 9 patients at week 48 compared to baseline. Seven out of nine patients achieved the target ASDAS score below 2.1 at week 48 (range 0.9–2, baseline ASDAS range 2.1–4.3), and for the remaining two patients, a ASDAS of 2.5 (baseline ASDAS 4.9) and 2.2 (baseline ASDAS 4.5) was reported at week 48 (Fig. 3A, *dashed red line indicates 2.1 cut-off*). During the week 24 and week 48 visits, another heparin blood sample was collected for analysis by MC. These longitudinal samples allowed us to monitor the changes in the cellular immunophenotype of the patients during treatment. Samples were processed and stained following the same protocol as baseline/control samples. To ensure we could track the initially defined clusters over time, we wished to apply the exact same clustering as was performed for the baseline vs control analysis. To this end, we trained an LDA classifier to “learn” the baseline vs control clustering and applied this to the newly obtained week 24 (*n*=8) and week 48 (*n*=9) samples. The LDA was able to assign the remaining samples, and only approximately 0–5% of the cells per sample remained unclassified (not shown). We focused our analysis on the clusters significantly different between baseline and control (9 clusters, Fig. 3B). In 5 out of 9 clusters, the frequency within week 48 samples changed significantly compared to baseline; these include cl10 CD4^+^ T_nai_ cells median 4.7% to 0.02% *p*=0.04, cl37 CD4^+^ T_em_ cells median 0.13% to 8.28% *p*=0.008, cl8 CD4^+^ T_cm_ cells median 3.2% to 0.02% *p*=0.008, cl39 B cells median 0.12% to 2.56% *p*=0.008, and cl5 CD38^+^ B cells median 2.52% to 0.64% *p*=0.02. In 4 of these clusters (cl10, cl37, cl8, cl39), the frequencies at week 48 were now comparable to controls. The frequency changes of these subsets coincided with decrease in ASDAS score, suggesting that these subsets are linked to disease activity or possibly targeted by the NSAID and/or DMARD treatment. Interestingly, one cluster (cl5: CD38^+^ B cells) significantly decreased at week 48 compared to baseline, but week 48 levels were still significantly higher compared to controls (median controls 0.08%; *p*=0.04). The patient showing the highest frequency of Cl5 at week 48 (2.5%) was also the patient with the highest ASDAS score at week 48, though the study size is too small to truly correlate cluster frequencies to ASDAS.

## Discussion

The goal of this inventory study was to evaluate to potential of MC in clinical trials and longitudinal studies in the context of axSpA. Our study identified differences in cellular immune phenotype between patients and HLA-B27^+^ age-matched controls. Our longitudinal samples showed changes over time in the immune profile of axSpA patients and additionally indicated that the immunophenotype largely normalized upon treatment when compared to HLA-B27^+^ controls. Based on our results, we were able to identify patterns within the cellular immunophenotype that changed upon treatment initiation and indicated that MC can represent a very valuable tool to investigate normalization of the cellular immunophenotype following treatment initiation. In doing so, the employment of deep cellular phenotyping in clinical trials could improve our knowledge of why treatment is effective or which cellular pathways are at play in axSpA. Moreover, to our knowledge, this is the first study implementing MC immuno-monitoring in an axSpA clinical trial to investigate baseline differences as well as changes during treatment.

Despite these insights, there are clear limitations to our study. First, we were not able to obtain follow-up samples of the healthy HLA-B27^+^ controls, as repeated PBMC sampling of HLA-B27^+^ controls is as of now not implemented in the SPACE study. Longitudinal control samples would have helped to map the natural individual changes on an immunological level and further filter which of the observed changes are indeed related to disease/treatment. Secondly, this is a relatively small study including 9 patients. Though we already observe strong differences in this inventory study, our cohort is too small to make assumptions on specific cellular subsets involved in axSpA pathogenesis or response to therapy. Furthermore, without replication, our results remain preliminary, and thereby caution should be taken with assigning subsets at play in axSpA pathogenesis and treatment responses. In this line of reasoning, it is of importance to obtain and analyze in situ material obtained from affected tissues as well. Although difficult from axial joints, synovial fluid from affected peripheral joints could represent a valuable source for studying cells along with PBMCs to further the understanding of axSpA. Our current set up does not allow to conclude if specific subsets may have migrated into the affected tissue as a possible explanation for low peripheral frequencies. In summary, future studies should include a larger patient cohort, preferably include in situ material, and aim to include multiple time points for controls to correct for possible natural individual changes in the immunophenotype.

Interestingly, our MC panel included markers dedicated to various lineages, cells of both the innate and adaptive immune system, and provided a broad immune profile of patients and changes observed during their treatment. Among these, CD27 expression by CD4 T cells seems connected to early axSpA, as nearly all CD27^+^ expressing CD4 T cells subsets are present in BL samples and absent in controls (Fig. 1A, B). Moreover, the CD27^+^ CD4 T cell subset cl10 was more frequent in BL axSpA and its frequency normalized during treatment. In our previous work, comparing two types of early untreated rheumatoid arthritis patients, we identified a somewhat similar CD27^+^ CD4 T cell subset [7]. At that time, the subset was not studied in more detail as distribution in both groups was similar. Perhaps this CD27^+^ CD4 subset is linked to a more general state of inflammation in rheumatic diseases, rather than an axSpA-specific process. Nonetheless, MC allows to study a large variety of markers simultaneously. Therefore, our study highlights the investigation of CD4 T cells and their development in early rheumatic disease, potentially using CD27 to identify subsets of interest in comparison to controls. Our study also highlighted the power of high-parameter unsupervised analysis as some of the findings are related to B cells, which are not commonly described to play a role in axSpA and are worth to further investigate on a larger scale. For example, Wilbrink et al. recently described that the frequency of CD27-CD38lowCD21low B cells is increased in axSpA compared to healthy donors [20]. Although our panel did not include CD21, making a direct comparison with the data presented by Wilbrink et al. challenging, the expression of CD27 and CD38 on B cells was analyzed. In this analysis, we did not detect an increase in CD27-CD38low B cells. Our data did indicate a different distribution of the B cell population between patients and controls, a distribution that normalized after start of treatment. Therefore, our data are in agreement with the notion put forward by Wilbrink et al. indicating that in the context of axSpA B cells could be of interest despite ruling “against prevailing dogma” [20]. As CD27+ T cells are important for proving helper support for B cells, our proof of concept study demonstrates the importance of studying multiple immune cells simultaneously to ensure a full view of immunological processes in early stages of axSpA and in relation to treatment effects. It would be interesting to monitor these subsets in axSpA, but also potentially other rheumatic diseases to help understand if the phenotypes observed are axSpA specific or linked to rheumatic inflammation in general.

Thus, with the pathophysiology of axSpA incompletely understood, our inventory study showed the value of deep cellular phenotyping when applied to a disease such as axSpA. Deep cellular phenotyping such as facilitated by MC allows for broad screening for surface markers as well as intracellular and intranuclear (phospho-) targets, allowing researchers to study both cell-cell communication as well as intracellular pathways on a high-parameter, multi-center level. Additionally, the possibility of freezing samples after MC staining allows more flexibility, especially for multi-site studies [21]. Studies investigating the cellular phenotype of axSpA could further help improve the search for biomarkers linked to effective treatment and thereby help to improve patient care and reduce disease-associated damage and progression.

## Supplementary information

ESM 1 (PDF 437 KB)

ESM 2 (PDF 564 KB)

ESM 3 (PDF 441 KB)

## Data Availability

The datasets used for the current study are available from the corresponding author upon reasonable request.
