# Supplementary material for: Spondyloarthritis mass cytometry immuno-monitoring: a proof of concept study in the tight-control and treat-to target TiCoSpA trial
Source: Clin Rheumatol. 2023 Jun 12;42(9):2387–96. doi: 10.1007/s10067-023-06637-1 (PMC10412466; doi:10.1007/s10067-023-06637-1)
Supplement: Supplementary file 1 — (PDF 437 KB) [file 10067_2023_6637_MOESM1_ESM.pdf]

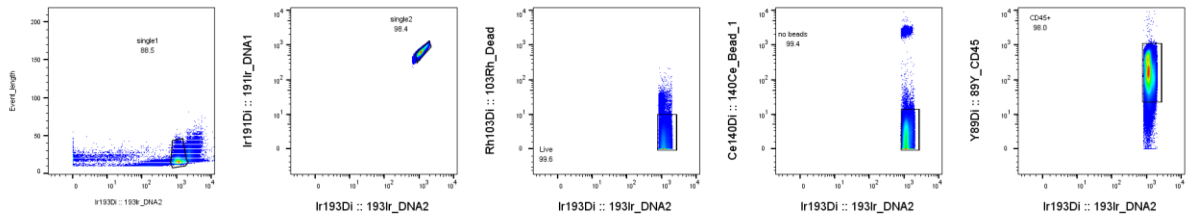

Supplementary Figure 1: Data clean up gating strategy

Data were normal normalized using EQ passport P13H2302 within the Fluidigm software. Gating of normalized files was performed following a sequential strategy starting with DNA2-^{193}Irridium vs Event length to remove the majority of debris and doublets, followed by DNA1-^{191}Irridium vs DNA2-^{193}Irridium to further define single cells. Live cells are defined as ^{103}Rhodium negative and beads are removed using ^{140}Cerium. A final CD45+ gate was applied and remaining events were exported into new FCS files for further downstream analysis.
