# Supplementary material for: Spondyloarthritis mass cytometry immuno-monitoring: a proof of concept study in the tight-control and treat-to target TiCoSpA trial
Source: Clin Rheumatol. 2023 Jun 12;42(9):2387–96. doi: 10.1007/s10067-023-06637-1 (PMC10412466; doi:10.1007/s10067-023-06637-1)
Supplement: Supplementary file 2 — (PDF 564 KB) [file 10067_2023_6637_MOESM2_ESM.pdf]

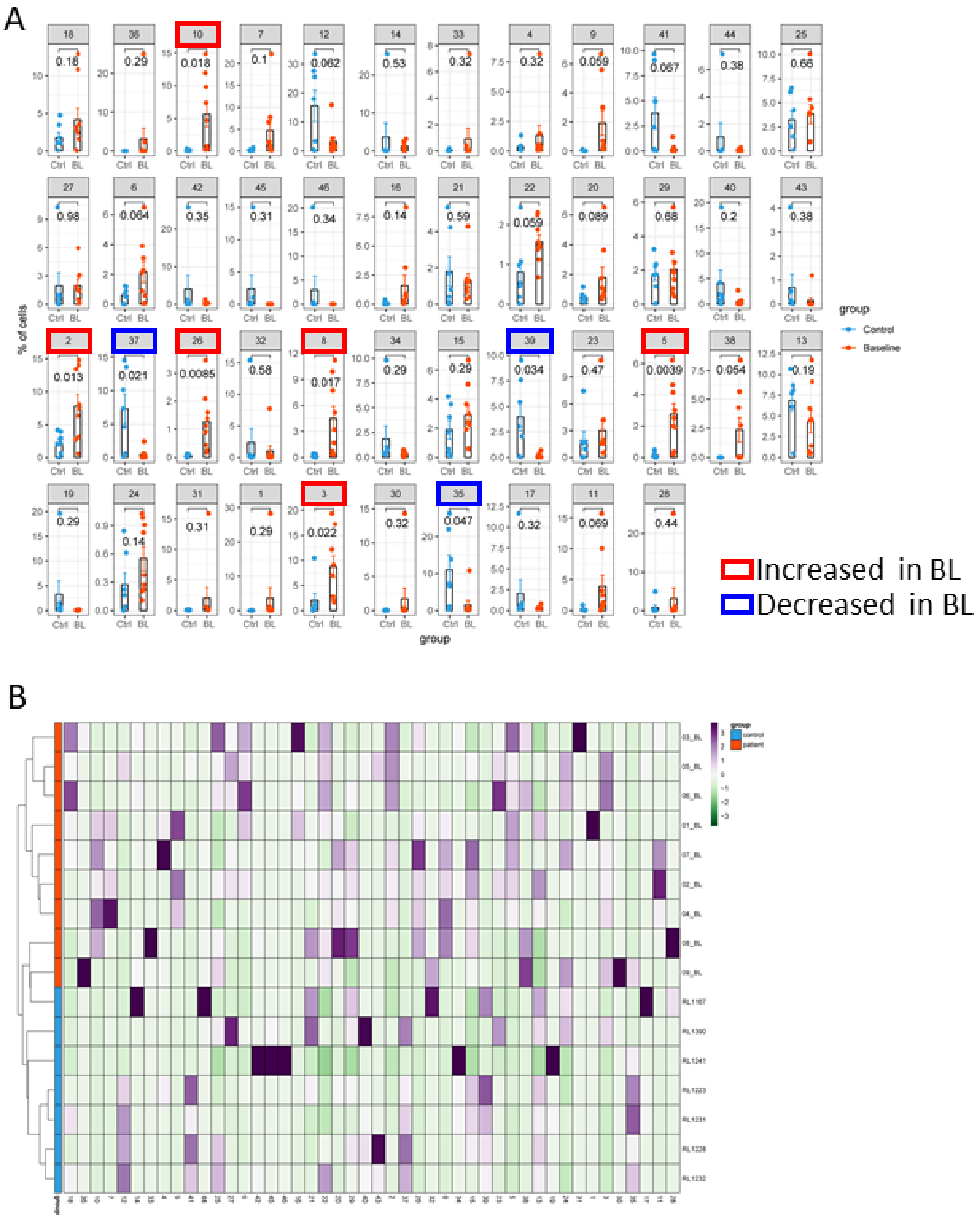

Supplementary Figure 2: Cytofast output

A) Cytofast output showing all clusters and their respective P-values. Colored boxes indicate the 9 clusters significantly different when comparing baseline patients to controls (RED increased at baseline; BLUE decreased at baseline). B) Cytofast output heatmap plotting frequencies of each cluster per sample: columns represent clusters and rows represent patient and control samples (RED patient; BLUE control). Ordering of rows is based on similarity between samples.
