# Supplementary material for: Spondyloarthritis mass cytometry immuno-monitoring: a proof of concept study in the tight-control and treat-to target TiCoSpA trial
Source: Clin Rheumatol. 2023 Jun 12;42(9):2387–96. doi: 10.1007/s10067-023-06637-1 (PMC10412466; doi:10.1007/s10067-023-06637-1)
Supplement: Supplementary file 3 — (PDF 441 KB) [file 10067_2023_6637_MOESM3_ESM.pdf]

Supplementary Table 1: Antibodies used for Mass Cytometry

Antigen	Tag	Clone	Company	Cat#
**CD45**	89Y	HI30	FDM	3089003B
**CD196**	141Pr	G034E3	FDM	3141003A
**CD19**	142Nd	HIB19	FDM	3142001B
**CD117**	143Nd	104D2	FDM	3143001B
**CD69**	144Nd	FN50	FDM	3144018B
**CD20**	145Nd	2H7	BioLegend*	302343
**CD8α**	146Nd	RPA-T8	FDM	3146001B
**CD303**	147Sm	201A	FDM	3147009B
**CD4**	148Nd	RPA-T4	BioLegend*	300541
**CD127**	149Sm	A019D5	FDM	3149011B
**Vα7.2**	150Nd	3C10	BioLegend*	351702
**CD123**	151Eu	6H6	FDM	3151001B
**TCRγδ**	152Sm	11F2	FDM	3152008B
**CD62L**	153Eu	DREG-56	FDM	3153004B
**CD3**	154Sm	UCHT1	FDM	3154003B
**CD45RA**	155Gd	HI100	FDM	3155011B
**IL-17A**	156Gd	BL168	BioLegend*	512331
**CD27**	158Gd	O323	BioLegend*	302839
**CD31**	159Tb	WM59	BioLegend*	303127
**CD28**	160Gd	CD28.2	FDM	3160003B
**CD14**	161Dy	M5E2	BioLegend*	301843
**CD56**	163Dy	NCAM16.2	FDM	3163007B
**CD161**	164Dy	HP-3G10	FDM	3164009B
**CD45RO**	165Ho	UCHL1	FDM	3165011B
**CD199**	168Er	L053E8	FDM	3168011A
**CD25**	169Tm	2A3	FDM	3169003B
**HLA-DR**	170Er	L243	FDM	3170013B
**FcεRI**	171Yb	AER-37	eBiosciences*	14-5899-82
**CD38**	172Yb	HIT2	FDM	3172007B
**CD94**	173Yb	HP-3D9	BD*	555887
**IL23R**	174Yb	218213	BioTechne*	MAB14001
**CD279**	175Lu	EH12.2H7	FDM	3175008B
**Ki-67**	176Yb	B56	BioLegend*	350523
**CD16**	209Bi	3G8	FDM	3209002B

* in-house conjugated at 100µg scale using a Maxpar© X8 antibody labeling kit (Fluidigm)
